# Bioaccessibility and Bioavailability of Diet Polyphenols and Their Modulation of Gut Microbiota

**DOI:** 10.3390/ijms24043813

**Published:** 2023-02-14

**Authors:** Tamara Lippolis, Miriam Cofano, Giusy Rita Caponio, Valentina De Nunzio, Maria Notarnicola

**Affiliations:** National Institute of Gastroenterology—IRCCS “Saverio de Bellis”, 70013 Castellana Grotte, Italy

**Keywords:** diet-derived antioxidants, heath, gut microbiota, micro-encapsulation, polyphenols

## Abstract

It is generally accepted that diet-derived polyphenols are bioactive compounds with several potentially beneficial effects on human health. In general, polyphenols have several chemical structures, and the most representative are flavonoids, phenolic acids, and stilbenes. It should be noted that the beneficial effects of polyphenols are closely related to their bioavailability and bioaccessibility, as many of them are rapidly metabolized after administration. Polyphenols—with a protective effect on the gastrointestinal tract—promote the maintenance of the eubiosis of the intestinal microbiota with protective effects against gastric and colon cancers. Thus, the benefits obtained from dietary supplementation of polyphenols would seem to be mediated by the gut microbiota. Taken at certain concentrations, polyphenols have been shown to positively modulate the bacterial component, increasing *Lactiplantibacillus* spp. and *Bifidobacterium* spp. involved in the protection of the intestinal barrier and decreasing *Clostridium* and *Fusobacterium*, which are negatively associated with human well-being. Based on the diet–microbiota–health axis, this review aims to describe the latest knowledge on the action of dietary polyphenols on human health through the activity of the gut microbiota and discusses micro-encapsulation of polyphenols as a strategy to improve the microbiota.

## 1. Introduction

### 1.1. Polyphenols and Their Therapeutic Application in Gastrointestinal Diseases

Polyphenols—organic chemical compounds with one or more phenolic rings with hydroxyl groups—are mainly present in foods such as fruits, vegetables, cereals, olives, pulses, chocolate, tea, coffee, wine, and grape pomace [1,2,3]. It is well-known that a large variety of phenolic compounds with complex chemical structures perform various beneficial, antioxidant, and protective effects in human pathologies. In fact, polyphenols can protect cell constituents from oxidative damage limiting the risk of various degenerative diseases associated with oxidative stress. Research literature strongly supports the role of polyphenols in a range of biological functions including anti-inflammatory, immunomodulatory, anticancer, antidiabetic, cardioprotective, neuroprotective, and gastroprotective properties [4,5,6,7]. In humans, polyphenols activate the antioxidant system through the upregulation of various endogenous antioxidants and the elimination of excess free radicals. Specifically, polyphenols are involved in the reduction of atherosclerotic plaques at the endothelial level by inhibiting the oxidation of low-density lipoproteins [8,9] and neutralizing free radicals responsible for aging, with antitumor effects [10,11]. Polyphenols exhibit not only antioxidant but also pro-oxidant effects, inducing apoptosis through the activation of caspases [6] and blocking cell proliferation [12,13]. Moreover, an important aspect concerns the chemo-preventive action of polyphenols consisting in affecting the tumor cells but not the normal cells [14]. Thus, polyphenols and their metabolites could be promising candidates for fighting various gastrointestinal diseases such as gastritis, gastric cancer, colorectal cancer, inflammatory bowel disease, and irritable bowel syndrome. Polyphenols and their active metabolites enhanced the production of short-chain fatty acids (SCFAs) and branched-chain amino acids (BCAAs) and could be useful in the treatment and prevention of various gastrointestinal disorders i.e., Crohn’s disease, ulcerative colitis, and colorectal cancer [15]. Results from in vitro and in vivo studies suggested that polyphenols, due to their antioxidant and anti-inflammatory effects, may modulate the colonic microbiota and contribute to the alleviation of symptoms of intestinal inflammation via the modulation of proinflammatory cytokines [16]. Moreover, a high intake of diet-derived polyphenols is associated with a lower risk of cancer, and new findings indicate that they also exhibit cancer-preventive effects [17]. In fact, it is well established that increased reactive oxygen species (ROS) production has been revealed in various types of cancers with numerous properties such as activating pro-tumorigenic signaling and enhancing cell survival and proliferation [18]. Experiments from a recent work highlighted that polyphenols from grape pomace are involved in antiproliferative and pro-apoptotic effects in HT29 and SW480 colorectal cancer cells [6]. Moreover, polyphenols modulate the immune system response and protect normal cells from free radical damage [19].

Based on the previous evidence, there is a bidirectional interaction between polyphenols and gut microbiota [20]. Since gut microbiota is a topic that is emerging as a key player in the relationship between dietary habits and health, it is important to study the effects involving the polyphenol–gut microbiota axis. Although scientific evidence extensively discusses the importance of the dietary intake of polyphenols in various diseases [21], this review focuses more on the modulation of polyphenols in dysbiosis conditions involved in gastrointestinal diseases through the regulation of gut microbiota. In addition, evidence from the main in vitro and in vivo studies on the beneficial microbial patterns of the gut microbiota in terms of the dose and format of phenolic compounds in specific foods were summarized. In particular, the potential physiological gastrointestinal effects of in vitro and in vivo studies focusing on phenolic compounds were reviewed. However, the beneficial effects of polyphenols are closely related to their bioavailability and bioaccessibility, thus it was assessed that gastrointestinal digestion of polyphenols improves their absorption and recovery index [2,22]. Considering the potential role of dietary polyphenols on human health, this review aims to describe the latest knowledge on the action of dietary polyphenols on human health through the activity of the gut microbiota and discusses micro-encapsulation of polyphenols as a new strategy to improve the microbiota. Indeed, polyphenols—with a protective effect on the gastrointestinal tract—promote the maintenance of eubiosis of the gut microbiota with protective effects against gastric and colon cancers. Therefore, the benefits obtained from dietary supplementation of polyphenols appear to be mediated by the gut microbiota.

### 1.2. Gut Microbiota and Diet-Derived Components

The human gastrointestinal tract harbors a unique and complex polymicrobial ecosystem made up of trillions of cells [23]. The gut microbiota is an additional organ that contributes to the nutrient metabolism of dietary components, influencing human health by producing harmful or beneficial metabolites, protecting against pathogens, modulating the immune system, and protecting against various diseases [24]. The composition of the intestinal microbiota is influenced by various factors such as genetics, age, stress conditions, medications, and diet. A strong correlation between microbiota composition and diet is demonstrated as a consequence of long-term dietary habits [25]. In this perspective, the macro- and micro-food components can influence the composition and metabolic pathways of the intestinal microbiota. Diet is the main carrier of microbes and, more importantly, acts as a source of nutrients for the oral and gastrointestinal microbiota. Thus, evidence has supported differences in the microbiome and metabolome based on dietary habits, particularly by comparing agrarian diets, such as the Mediterranean and Okinawan diets, with the Western diet, which is primarily characterized by high fat, refined sugars, and animal proteins and bottom-fermented fibers [26].

Beneficial dietary effects derive from the intake of specific nutrients and are introduced in a sufficient quantity to improve the health of the host, acting as functional foods. Due to a not universally accepted definition of functional foods, various definitions have been given over time underling that the common factor is the health benefit on the host’s physiology [27].

A prebiotic is a non-digestible product for the human body that positively influences the host by selectively stimulating the growth and/or activity of one or a limited number of bacterial species already resident in the colon. A food ingredient, to be classified as a prebiotic, must not be hydrolyzed or absorbed in the upper gastrointestinal tract, must serve as a selective substrate for one or a limited number of potentially beneficial commensal bacteria in the colon, stimulating their growth or activating their metabolism, and finally, must be capable of altering the colonic microflora toward a presumably more favorable composition in terms of host health. Of all these characteristics, those specific to prebiotics are selectivity and fermentation in a mixed culture environment. The first compounds studied as prebiotics were those that promote the growth of lactic acid-producing microorganisms, such as lactulose, which is used in infant formula to increase lactobacilli in the gut [28]. Prebiotics include dietary fibers such as arabinoxylan—a nonstarch polysaccharide found in many cereals—some polysaccharides found in algae and microalgae, and oligosaccharides, although only fructans such as inulin and galacto-oligosaccharides fully meet the criteria established for classification as prebiotics. The benefits of prebiotics are mediated by their ability to modify the gut microbiota and especially to selectively modulate specific strains, stimulating the growth of beneficial species already residing in the colon. Because of their chemical structure and the resulting inability of the host to digest them, prebiotics are fermented directly in the colon by endogenous bacteria with SCFAs, resulting in a lower pH. Through this process, they can exert anti-inflammatory effects, such as stimulating an increase in regulatory T cells and a reduction in interferon [28]. Prebiotics can also inhibit the adhesion of pathogens to the intestinal epithelium, preventing their passage through the epithelium [29,30].

The most fermented dietary fibers by the gut microbiota are fructo-oligosaccharides, also known as FOSs, including inulin, galacto-oligosaccharides or GOSs, human milk oligosaccharides, isomalto-oligosaccharides, and xylans found in various foods, including asparagus, soy, artichoke, cucumber, wheat, honey, and breast milk [31]. Therefore, due to the high fiber content, the consumption of fruits and vegetables is associated with greater microbial richness, in particular, with beneficial saccharolytic bacterial patterns, such as lactobacilli and bifidobacteria, with known probiotic activity. In fact, probiotics are the main microbial patterns capable of metabolizing fibers into SCFAs. In turn, SCFAs have a broad spectrum of beneficial and healthful effects, such as energy substrates for colonocytes, improvement of the intestinal barrier function through mucin synthesis, and improvement of the immune system.

In specific pathologies, such as celiac disease and non-celiac gluten sensitivity, in which gluten causes adverse gastrointestinal symptoms, the gluten-free diet remains the only therapy adopted to date [32]. However, several trials have shown that strict adherence to a gluten-free diet leads to an unbalanced microbial composition [33]. The reason is linked to the absence of wheat in gluten-free baked goods which leads to a critical shortage of fructans. This class of fibers, similar to other prebiotics, plays a fundamental role in maintaining a eubiotic microbiota, in fact, previous studies have shown that even in healthy subjects, a gluten-free diet favored the onset of symptoms of intestinal relief only after switching to a normal diet again [34]. In recent years, a novel food considered a functional food has taken place, namely algae. Seaweeds—traditionally consumed as food or as medicinal herbs—have high nutritional values, due to their high protein content, and pharmaceutical values [35]. Therefore, among the different ways existing to manipulate the intestinal microbiota, the active components of algae are included. The consumption of seaweed applied to the physiopathology of type 2 diabetes mellitus can determine positive effects such as a reduction in hyperglycemia, hyperinsulinemia, and insulin resistance due to the high fiber content [36]. Furthermore, positive effects are also linked to the reduction in oxidative stress, due to the high quantity of antioxidants, and the energy intake from carbohydrates and fats, through the modulation of the microbiota. In addition to seaweed, other vegetables and fruits are also a source of micronutrients, such as vitamins and polyphenols. Among the main foods with a known high content of polyphenols are wine, green tea, grapes, red fruits, and coffee.

**Figure 1:**
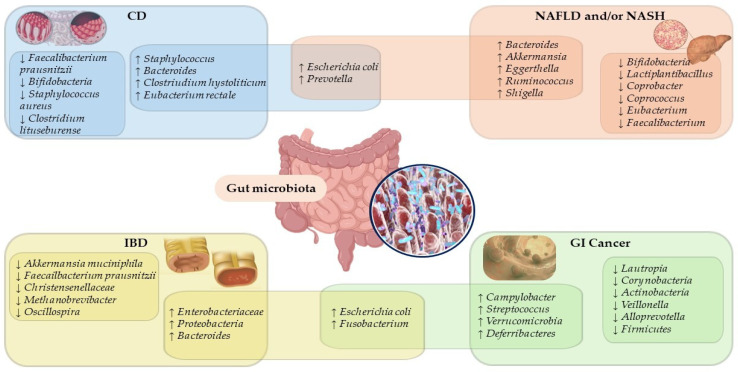
Overlapping microbiota species and genera signatures in gastrointestinal disease. Abbreviation: CD, celiac disease; GI, gastrointestinal; IBD, inflammatory bowel disease; NAFLD, non-alcoholic fatty liver disease; NASH, non-alcoholic steatohepatitis; ↓, decrease; ↑, increase.

Since polyphenol-bioactive compounds from the diet and the health of the gut microbiota are closely related, maintaining a eubiotic state in the gut microbial ecosystem is essential to prevent a microbial imbalance related to various gastrointestinal diseases (Figure 1) [37,38,39,40,41,42].

## 2. Diet-Derived Polyphenols: Healthy Outcomes

**Figure 2:**
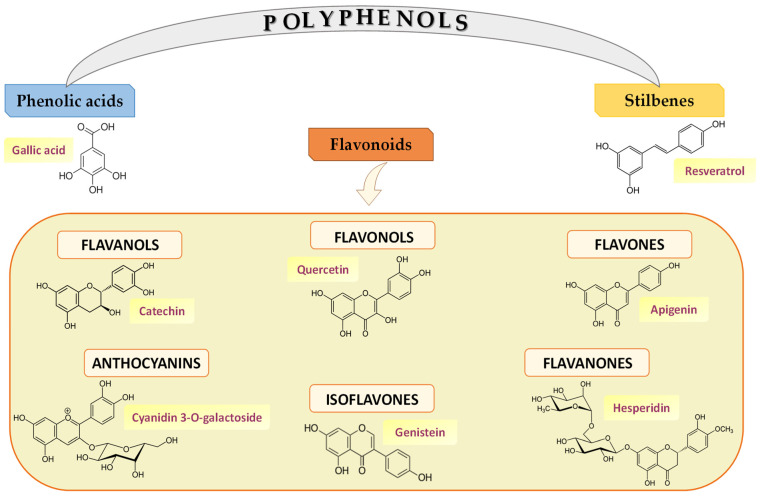
Polyphenol classes and chemical structures of some of their main compounds.

Polyphenols—constituents of plant-derived foods—include a wide variety of molecules divided into different classes based on their structure. Figure 2 illustrates the structures of the main polyphenolic compounds mentioned below belonging to flavonoids, phenolic acids, and stilbenes [43,44]. Flavonoids contain a common carbonaceous skeleton of diphenylpropanes, two benzene rings (A and B ring) joined by a three-carbon straight chain. The central chain can form a closed pyranic ring (C ring) with one of the benzene rings. Generally, flavonoids are divided into six different subclasses: flavonols, flavones, flavanones, isoflavones, anthocyanins, and flavanols.

Flavonols—including quercetin and kaempferol—are the most representative compounds of foods such as onions, cabbage, leeks, broccoli, blueberries, tea, and red wine. Studies in the literature have shown that kaempferol has strong cellular antioxidant capacity by eliminating ROS accumulation and shows antiproliferative activity against various human cancer cells and induces apoptosis of MCF-7 cells through the mitochondrial pathway [45]. Additionally, quercetin showed in vitro antioxidant activities and is involved in the removal of free radicals [46] and the regulation of metal ions such as, for example, Cu^2+^ and Fe^2+^ ions. Tang et al. 2014 [47] reported that C57BL/6J mice with alcohol-induced liver disease treated with quercetin showed inhibition in Fe^2+^-induced lipid peroxidation by going on to bind and inhibit the action of the ion itself. In addition, quercetin also demonstrated antitumor action by significantly modulating the cell cycle, promoting cell apoptosis, and inhibiting the generation of new blood vessels in several cancer cell lines [48,49,50].

In contrast to flavonols, flavones represent the least representative class of flavonoids and are mainly found in parsley, celery, and the skin of some fruits. Research in the literature has highlighted the role of apigenin and its glycosides in influencing the state of intestinal health in the prevention of some types of cancer [51]. Moreover, flavanones are found in tomatoes and some aromatic plants, such as mint, and in very high concentrations in citrus fruits, such as in the case of hesperidin. In fact, orange juice contains up to 470–761 mg/L of hesperidin [52], although most flavanones are concentrated in the whitish part of the fruit, i.e., the albedo. Studies on streptozotocin-induced diabetic rats showed that hesperidin has potential anti-hyperglycemic activity by reducing plasma glucose levels in a dose-dependent manner and improving glycogen content in the liver tissue through restoring the activities of the enzyme’s glycogen synthase and glycogen phosphorylase [53]. In addition, it was demonstrated that hesperidin significantly reduced HepG2 cell proliferation and induced apoptosis by activating the intrinsic caspase-3-dependent pathway through upregulation of the proapoptotic protein Bax [54]. The class of flavonoids also includes isoflavones, which are structurally similar to estrogens and classified as phytoestrogens capable of binding to their receptors [55]. Isoflavones are mainly found in legumes, especially soybeans [56]. Genistein, mostly found in these foods, exhibits antioxidant, anti-inflammatory, anti-angiogenic, pro-apoptotic, and antiproliferative activities, conferring its chemo-preventive and chemotherapeutic potential [57].

Anthocyanins are water-soluble pigments that are responsible for the red, blue, and purple coloration of fruits, vegetables, and other plants; thus, they are mostly found in red wine, some varieties of cereals, and some vegetables (cabbage, beans, onions, radishes) and fruits. Anthocyanins are mainly found in the skin, except in some red fruits such as cherries and strawberries, where they are also present in the pulp. Importantly, among them stand out malvidin-3-galactoside (M3G) and Cyanidin 3-O-galactoside (Cy3Gal). M3G suppressed the proliferation, polarization, migration, and invasion of HepG2 cells in vitro by regulating the protein expression of cyclins D1, B, E and cleaved caspases-3 and caspase-3, Bax, p-JNK and p-p38, and by activating PTEN with decreased levels of p-AKT and MMP-2 and -9; while in vivo, M3G promoted apoptosis of liver cancer cells [58]. Cy3Gal, and its combination with other polyphenols, also had numerous effects on human health, including antioxidant, anti-inflammatory, antitumor, antidiabetic, antitoxic, cardiovascular, and neuronal-level properties [59]. So, these results suggest that both M3G and Cy3Gal, used as adjuvant ingredients or nutritional supplements, are involved in preventing cancer development with beneficial effects on human health.

Finally, flavonoids include flavanols, which are present in both monomers (catechins) and polymer (proanthocyanidins) forms. The main catechins found in fruit are catechin and epicatechin, while gallocatechin, epigallocatechin, and epigallocatechin gallate are mainly found in tea. Proanthocyanidins, also known as condensed tannins, are dimers, oligomers, and polymers of catechins and are responsible for the astringent character of fruits, drinks, and the bitterness of chocolate [60].

Another prominent class of polyphenols consists of phenolic acids divided into two classes, namely benzoic acid derivatives and cinnamic acid derivatives. Phenolic acids are types of aromatic acid compounds including substances containing an aromatic ring and a benzene ring with one or more hydroxide groups including functional derivatives [61]. Hydroxybenzoic acids, such as gallic acid (GA) and protocatechuic acid, are present in low concentrations in a few plants consumed by humans, except for some red fruits such as, for example, blackberries. Among beverages high in GA is tea; in particular, tea leaves contain up to 4.5 g/kg fresh weight [62], while protocatechuic acid is mostly found in raspberries and olive oil [63]. Moreover, hydroxycinnamic acids include coumaric, caffeic, and ferulic acids.

Stilbenes are characterized by the presence of a 1,2-diphenylethylene nucleus with hydroxyl substituted on the aromatic rings and exist in the form of monomers or oligomers [64]. The main compound representative of stilbenes is resveratrol, mainly concentrated in grapes, berries, and peanuts. Several data demonstrated the anticarcinogenic effects of resveratrol [65] particularly colorectal and obesity-related effects [66].

Polyphenols in green tea induced an increase in SCFAs-producing bacteria, such as *Lachnospiraceae*, *Ruminococcaceae*, and *Bifidobacteriaceae*, and a reduction in the abundance of oral and fecal *Fusobacterium* in healthy subjects [67]. Therefore, the production of SCFAs following polyphenol intake is associated with an improvement in some gastrointestinal diseases [68,69]. Indeed, scientific evidence has pointed out that the increase in SCFAs-producing bacterial taxa is involved in the reduction in intestinal inflammation in subjects with colorectal cancer [70,71].

## 3. Polyphenols: Bioaccessibility and Bioavailability

To evaluate the efficacy of polyphenols in disease prevention and the improvement of human health, it is important to understand the nature and distribution of these compounds in the diet in order to determine the most promising polyphenols in terms of bioavailability, which in turn depends on the relative content of compounds released from the food matrix along the digestive system (bioaccessibility), digestive stability, and efficiency of the transepithelial passage (intestinal absorption) [72,73,74]. In fact, the biological function of each polyphenol is related to its metabolism (biotransformation), absorption (intestinal barrier), and utilization rate (bioavailability) and is influenced by various factors such as structure (binding with prosthetic groups), chemical interactions (with other macromolecules), and biotransformation process [75].

Because polyphenols can have different chemical structures, it is not immediately possible to quantify their exact content in foods. Moreover, the beneficial action of polyphenols on human health depends not only on their content in foods but also on other factors such as their stability, microbiota, and digestive enzymes. Scientific evidence points out that among polyphenols, isoflavones are the most bioavailable followed by phenolic acids, flavanols, flavanones, and flavonols, and the last mentioned are anthocyanins and proanthocyanidins [76]. The bioavailability of flavonoids—which are hydrophilic compounds—is quite low because they are not well absorbed in the gut, so they cannot be used as nutraceuticals due to their low bioactivity. A viable strategy to improve their bioavailability is using microencapsulation and microemulsions of flavonoids. Therefore, it is important to consider that the bioaccessibility of polyphenols is not necessarily positively correlated with high concentrations of phenolic compounds in food matrices [77,78]. Important aspects concern the chemical structure of polyphenols and the diversity of species and genera in the gut microbiota, which allows for the production of certain enzymes involved in the biotransformation of polyphenols, such as deglycosylation [79,80]. In summary, bioavailability is profoundly influenced by the great diversity of polyphenol chemical structures, and a high polyphenol content in foods does not necessarily correlate with high bioavailability. In addition, biotransformations promoted by the gut microbiota affect bioavailability, which in turn is influenced by absorption and metabolism.

**Figure 3:**
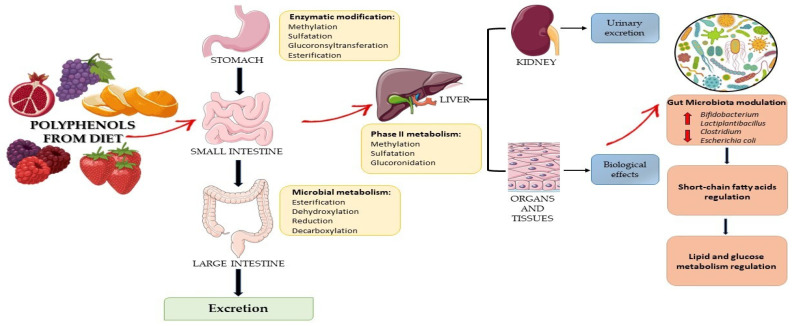
Effects of dietary polyphenols on gut microbiota, their metabolites, and health benefits.

Small polyphenols can be directly adsorbed into the small intestine, while complex polyphenols remain undigested up to the large intestine. At this level, they are converted into low-molecular-weight metabolites by the gut microbial communities, which are bioavailable for host adsorption through methylation, sulfation, and glucuronidation reactions. Secondary metabolites derived from the microbial metabolism of polyphenols act as prebiotic-like molecules that, in turn, can modulate the growth of specific bacterial strains (Figure 3) [81]. Polyphenols interact with other food components in the intestine by binding to macromolecules such as fibers and forming chemical complexes and colloidal structures that reduce or enhance their bioavailability [82]. To the best knowledge, molecules absorbed from the small intestine are aglycones; however, most polyphenols are present in food in the form of esters, glycosides, or polymers that cannot be absorbed in their native form. In fact, these molecules must undergo hydrolysis by intestinal enzymes to facilitate their absorption. As shown in Figure 3, during absorption, polyphenols, through the processes of methylation, sulfation, and glucuronidation, are conjugated in the small intestine and liver, increasing their hydrophilicity and facilitating their urinary elimination [82]. Moreover, the impact of polyphenols on microbiota maintains a healthy microbiome that, in turn, shows suppressive effects on the progress of life-style related diseases, such as diabetes. Additionally, some classes of polyphenols, bio-transformed by microbiota metabolism, showed an anti-inflammatory effect delaying the onset and/or progression of different gastrointestinal pathologies, including ulcerative colitis. Thus, polyphenols are involved in the interaction with the gut microbiome and can operate as prebiotics and enhance the production of various beneficial microorganisms in the host’s gut, positively modulating the gut microbiota. In fact, these compounds not digested by human digestive enzymes are used as a substrate for selective microorganisms that confer human health benefits. The gut microbiota plays an important role in regulating the production of SCFAs, BCAAs, and vitamins and positively modulates lipid and glucose metabolism, thus promoting the overall health status of the host [83,84]. Moreover, it was confirmed that dietary supplementation with polyphenols can significantly enhance the production of health-beneficial bacterial species such as *Bifidobacterium* and *Lactiplantibacillus*, as well as suppress the production of damaging species such as *Clostridium* and *Escherichia coli (E. coli)* [85].

## 4. Polyphenols–Microbiota–Health Axis

It is well-known that gut microbiota dysbiosis—described as an altered microbial community with an increased population of pathogenic bacteria—can lead to pathological conditions. In fact, a change in terms of microbial diversity, bacterial functionality, and the presence of beneficial inhabitants often correlates with an increase in harmful ones, which seems to be implicated in cases of obesity, type 2 diabetes, inflammatory bowel disease (IBD), and colorectal cancer [86]. Diet is one of the factors that can positively modulate intestinal microbiota composition. Previous works showed how the production of fresh pasta using inulin—a soluble fiber—instead of durum wheat semolina significantly reduced *E. coli* cell density and increased prebiotic growth [87]. Even the polyphenols ingested through the diet can interact with intestinal microorganisms and determine a change toward a healthier profile. The mechanisms through which polyphenols modulate the microbiota are still unclear but in general, they can intervene both directly and indirectly. They can directly stimulate or inhibit the growth of some bacteria. In the first case, resistance is strongly associated with the ability of bacteria to metabolize these compounds, while in the second case, inhibition is related to the antimicrobial capabilities of these compounds [88,89]. In this sense, several works report the selective bactericidal effect of polyphenols against specific bacteria [88]. Recently, the antimicrobial effect of grape pomace polyphenols in combination with a probiotic, *Lactiplantibacillus plantarum*, on the growth of pathogenic microorganisms such as *E. coli*, *Bacillus megaterium*, and *Listeria monocytogenes* has been demonstrated [2]. Different polyphenols introduced with the diet such as gallic acid, hesperedin, and naringin can modulate the intestinal microbiota through antimicrobial and prebiotic actions (Table 1). Regarding GA, previous studies demonstrated many biological properties, including antioxidant, antitumor, anti-inflammatory, and antimicrobial properties, and recent studies have shown how GA and its metabolites improve the activity of the gut microbiome by modulating immune responses [49]. GA inhibited the development of metastasis in gastric adenocarcinoma cells by inhibiting the Ras/PI3K/AKT signaling pathway [90], reduced the viability of human colon cancer cells by suppressing cell proliferation, and was involved in the regulation of the NF-κB, AP-1, STAT-1, and OCT-1 signaling pathways [91]. In addition, recent studies have evaluated the effects of GA on the gut microbiota. In general, dietary intake components are used by the gut microbiota to produce energy and metabolites. These metabolites enter the bloodstream and influence gut activity and the immune system [92]. A recent study evaluated the effects of GA, in a mouse model with ulcerative colitis, on reducing dysbiosis of the gut microbiota. Indeed, following GA intake, an increase in the *Lactobacillaceae* and *Prevotellaceae* families and a reduction in some pathogenic bacteria such as *Firmicutes* and *Proteobacteria* were recorded [93,94,95]. Furthermore, GA is able to alleviate the environmental stress of beagle puppies, which is considered an excellent model for the study of the human microbiota due to its high similarities with the human one [96], rebalancing the state of intestinal health through an increase in *Lactiplantibacillus* and *Faecalibaculum* and a reduction in *Escherichia*, *Shigella*, and *Clostridium*. Indeed, exposure to environmental stress is known to cause a disruption in the intestinal barrier, increased inflammatory responses, and intestinal dysbiosis [97].

Other polyphenols with positive activities on the gut microbiota are those contained in orange juice. Hesperidin and naringin, in healthy women, not only improved blood biochemical parameters (glucose, low-density lipoprotein cholesterol, and insulin sensitivity) but more importantly, positively regulated the composition and metabolic activity of the microbiota by increasing the population of *Bifidobacterium* spp. and *Lactiplantibacillus* spp. and increasing SCFA production. These results suggest that orange consumption improves the gut microbiota and its metabolites [98]. The composition and activity of the microbiota were also tested on an in vitro model of the colon using the administration of a citrus extract with a high content of hesperedin and naringin, which observed an increase in beneficial bacteria and a reduction in harmful ones [99]. A study conducted on a model of hepatic steatosis in C57BL/6J mice fed a high-fat diet (HFD) in addition to resveratrol, rich in red grape skin [100], resulted in a change in the composition of the gut microbiota by going on to reverse HFD-induced dysbiosis with an increase in the abundance of *Bacteroidetes* and a decrease in *Firmicutes* and *Proteobacteria*, and by going on to reduce the parameters related to hepatic steatosis. Therefore, this study showed that resveratrol improves hepatic steatosis in HFD mice by positively modulating the gut microbiota [101].

A study conducted on rats with streptozotocin-induced diabetic peripheral neuropathy (DPN) showed how quercetin intake improved both neuropathy status and intestinal dysbiosis in DPN rats by decreasing four potential pathogenic species (f_*Porphyromonadaceae*, f_*Oxalobacteraceae*, g_*Oxalobacter* and g_*Klebsiella*) associated with DPN phenotypes and increased ROS and by enriching two prebiotic species (p_*Actinobacteria* and c_*Actinobacteria*) [102]. Thus, polyphenols exhibit prebiotic effects by increasing the beneficial bacteria abundance and inhibiting harmful ones, thus improving the status of some diseases [103,104]. For example, polyphenols contained in grape pomace extracts and seeds positively altered the gut microbiota of mice fed a high-fat diet by significantly increasing the relative abundance of *Prevotella* and reducing the relative abundance of *Streptococcus* [105], while proanthocyanidin extract from grape seeds in the same mouse model has been shown to have a potential prebiotic effect by positively regulating the growth of *Roseburia, Prevotella,* and *Clostridium XIVa* [106].

Red wine polyphenols in patients with obesity-associated metabolic syndrome also resulted in a significant increase in the number of fecal bifidobacteria and lactobacilli that are important in protecting the intestinal barrier at the expense of harmful LPS-producing bacteria such as *E. coli* and *Enterobacter cloacae* [107]. Furthermore, in healthy adults, red wine intake resulted in increased fecal concentrations of *Bifidobacterium*, *Enterococcus*, and *Eggerthella lenta* [108], although in vitro studies have shown inhibition of *Enterococcus* and *Eggerthella lenta* bacterial groups by GA and resveratrol metabolites [109].

At the basis of this direct antimicrobial action, there could be several molecular mechanisms: it has been demonstrated that subjects with IBD had an increase in *E. coli* caused in turn by an increase in adhesins, which facilitate the adhesion of bacteria to the surface of the intestinal epithelium [110]. Some phenolic compounds, such as resveratrol, have been able to inhibit the growth of *E. coli* and other bacteria harmful to human health, reducing the cell adhesion between these and the intestinal epithelium [111,112]. Furthermore, some polyphenols can exert their antimicrobial action by interacting with bacterial proteins and inhibiting bacterial nucleic acid synthesis, modifying the integrity and synthesis of the cell wall, altering the functionality and fluidity of the bacterial cell membrane, modulating the metabolism cell, inhibiting biofilm formation and *quorum sensing*, and chelated metals such as iron, copper, and zinc that are important for bacterial metabolism [113,114,115]. Cranberry polyphenol extract was implicated in the downregulation of genes coding for outer membrane proteins in *E. coli O157:H7*, with bactericidal effects against *Salmonella* strains, by reducing the expression of virulence genes and of those implicated in the cell wall and membrane biogenesis and acting as inhibitors of *quorum sensing* [116,117,118,119]. In addition to direct antimicrobial actions, polyphenols can also act indirectly: some scientific evidence has reported how some phenolic metabolites are able to influence the growth of some bacteria which, in turn, modulate the development of others [89,120,121]. Furthermore, polyphenols can also have a prebiotic effect by stimulating the development of beneficial bacteria through various mechanisms of action, such as providing the microbiota with carbon sources, acting as electronic acceptors, and generating proton motive forces during their metabolization [122].

**Table 1:** Main studies on the impacts of polyphenols on the gut microbiota.

**Polyphenols**	**Foods**	**Dose**	**Model**	**Main Findings**	**References**
GA	Plants, black-berries, tea	10 mg/kg BD for 7 days	DSS-induced colitis in mice	↑ *Lactobacillaceae*, *Prevotellaceae* ↓ *Firmicutes*, *Proteobacteria*	[93]
		500 mg/kg for 1 week	Dog puppies exposed to multiple environmental stressors	↑ *Lactiplantibacillus*, *Faecalibaculum* ↓ *Escherichia*, *Shigella*, *Clostridium*	[97]
HSP, NAR	Citrus fruits	300 mL/day OJ for 2 months	Healthy female	↑ *Bifidobacterium* spp., *Lactiplantibacillus* spp.	[98]
		250–350 mg/day CFE for 3 days	In vitro model of colon	↑ *Roseburia, Eubacterium ramulus,* *Bacteroides eggerthii* ↓ *Firmicutes*	[99]
RES	Grapes, berries, peanuts	4 g/kg for 12 weeks	HFD-induced hepatic steatosis in C57BL/6J mice	↑ *Bacteroidetes* ↓ *Firmicutes*, *Proteobacteria*	[101]
		60 mg/kg for 5 weeks	HFD-induced hyperglycemia in C57BL/6J mice	↓ *Alistipes*, *Clostridium*	[123]
QRC	Onions, cabbage, leeks, broccoli, blue-berries, tea, red wine	50 mg/kg BD for 12 weeks	STZ-induced diabetic peripheral neuropathy in rats	↑ f_*Porphyromonadaceae*, f_ *Oxalobacteraceae*, g_ *Oxalobacter*, g_ *Klebsiella* ↓ p_*Actinobacteria*, c_ *Actinobacteria*	[102]
		100 μL/10 g BD for 6 weeks	MSG-induced abdominal obese C57BL/6J mice	↓ *Firmicutes*, *Lachnospiraceae*, *Ruminicoccaceae* ↑ *Bacteroidetes*	[124]
GPE, GSE	Grape pomace	200 mg/kg BD for 7 days	HDF in C57BL/6J mice	↑ *Prevotella* ↓ *Streptococcus*	[105]
		300 mg/kg BD/day for 7 weeks	HFD-induced obesity in mice	↑ *Roseburia*, *Prevotella*, *Clostridium XIVa*	[106]
RWPs	Red wine	272 mL/day	Obesity-associated metabolic syndrome in adult	↑ Bifidobacteria and lactobacilli ↓ *Escherichia coli*, *Enterobacter cloacae*	[107]
		272 mL/day	Healthy adults	↑ *Bifidobacterium*, *Enterococcus*, *Eggerthella lenta*	[108]
GTP	Green tea	400 mL/day for 2 weeks	Healthy adults	↑ *Lachnospiraceae*, *Ruminococcaceae*, *Bifidobacteriacea* ↓ *Fusobacterium*	[67]
		0.67 mg/mL for 10 days	In vitro model of colon	↓ *Firmicutes* ↑ *Ruminococcaceae*	[125]

Abbreviations: BD, body weight; CFE, citrus fruit extract; DSS, dextran sodium sulphate; HFD, high fat diet; HSP, hesperidin; GA, gallic acid; GPE, grape pomace extracts polyphenols; GSE seed extracts polyphenols; GTP, green tea polyphenols; NAR, naringine; OJ, orange juice; QRC, quercetin RES, resveratrol; RWPs, red wine polyphenols; STZ, streptozotocin. ↓, decrease; ↑, increase.

## 5. Micro-Encapsulation of Polyphenols and New Strategies for Microbiota Improvement

Bioactive components of the diet, such as polyphenols, play a key role in modulating the gut microbiota. Today, growing evidence has shown that the gut microbiota can use these components to produce bioactive metabolites of phenolic acids. In fact, several metabolites show promising effects in the prevention and treatment of several diseases. In addition, scientific research has shown that polyphenol supplementation can modulate the growth and virulence properties of the gut microbiota to prevent and manage chronic diseases [126].

However, some polyphenols are insoluble in water and unstable, with low oral bioavailability, limiting their biological activities and hindering the process of studying the association between polyphenols and gut microbiota [127]. To better understand their impact on human health, encapsulation technologies have emerged rapidly in the gut microbiota scenario. Microencapsulation allows the protection of the biochemical functionality of a wide range of compounds by incorporating them into a protective matrix. Indeed, encapsulation increases the water solubility of polyphenols, protects them from unfavorable conditions during the digestive process, and releases them into targeted areas by modulating the gut microbiota [128].

To achieve good encapsulation efficiency, it is of primary importance to define encapsulating agents. There is a well-known and huge range of different materials used as encapsulating agents, which can be natural, semisynthetic, or synthetic polymers [129]. Various wall materials have been used over the years, including yeast cells, β-cyclodextrins, mixtures of alginate and chitosan, gelatin, maltodextrins, proteins, gum arabic, mesquite gum, xanthan, and inulin [130,131,132].

In particular, starch—composed of amylose and amylopectin—is safe, cheap, and easy to obtain, and it is suitable for encapsulation because of its biocompatibility and nontoxicity [133]. For these reasons, it can be modified with physical, chemical, and/or enzymatic approaches to achieve desired properties and encapsulate a range of hydrophilic and hydrophobic bioactive compounds such as polyphenols [134]. Accordingly, the encapsulation of polyphenols with starch could promote positive effects on gastrointestinal diseases playing an important role in modulating the gut microbiota. Several food ingredients have been successfully encapsulated in starch-based systems with optimal results [135]. Resistant starch could also efficiently deliver and release polyphenols at specific sites such as local treatment of colonic dysfunction to control the right dose of polyphenols to be administered [136]. Various resistant starch systems suitable for the encapsulation of polyphenolic substances, such as native starch [137], are available. In general, starch is widely processed using chemical approaches such as physicochemical methods and enzymatic hydrolysis [138]. Acetylation, esterification, and phosphorylation are common chemical modifications in which hydrophilic native starch is converted to hydrophobic starch derivatives. Recently, starch modified with octenyl succinic anhydride (OSA) in the amorphous form has been used in the encapsulation of hydrophobic polyphenols [139]. OSA-modified waxy corn starch could interact with tea polyphenols and tea catechins released gradually during in vivo digestion [140]. Enzymes including α-amylase, pullulanase, glucanotransferase, and others are frequently used for the preparation of branched starch or type III resistant starch [141]. Pullulanase-treated starch nanoparticles improved the stability and antioxidant activity of curcumin, and the encapsulation efficiency achieved was up to 92.49% [142]. Rice starch modified with 4-α-glucanotransferase, used to encapsulate curcumin, greatly improved its solubility, stability, and bioaccessibility.

Therefore, recent studies on polyphenols encapsulation using starch have shown that it controls the number of polyphenols released in the upper digestive tract potentially preventing the polyphenols from reaching the lower digestive tract. Thus, the encapsulation of polyphenols using starch can positively influence the abundance and composition of the gut microbiota with a significant role in human health.

## 6. Conclusions and Future Perspectives

Diet is a major driver of the composition and, especially, metabolic activities of the human gut microbiota. Particularly, polyphenols may exert anti-inflammatory, antioxidant, anti-cancer, and anti-diabetic activities by positively modulating the gut microbiota. Dietary macro- and micronutrients seem to be the main drivers of the metabolic pathways of the oral and intestinal microbiota affecting, in turn, the fecal, urinary, and blood metabolomes. Once normalized, the resilience of the microbiome, defined as the tolerance to perturbation, and the predisposition to a disease state could be weakened. Understanding bioactive molecules from the diet such as polyphenols, that underlie compositional and functional changes, allows us to design personalized therapies that target the gut microbiota. Nowadays, increasingly sophisticated and innovative strategies are available, such as the encapsulation of starch, to convey polyphenols, keeping the effectiveness of the molecules intact and increasing their absorption capacity in the body. Thus, resistant starch not only serves as a promising transporter but also as a prebiotic by influencing the abundance and composition of the gut microbiota. However, further studies are necessary to fully describe the complex food–gut human axis, and considering the key role of gut microbiota on human health, emerging dietary treatments are not only economical but also offer a targetable and non-invasive approach for treating chronic diseases.

## Data Availability

Data are contained within this article.
